# Deciphering the biodesulfurization potential of two novel *Rhodococcus* isolates from a unique Greek environment

**DOI:** 10.3934/microbiol.2022032

**Published:** 2022-12-15

**Authors:** Panayiotis D. Glekas, Olga Martzoukou, Maria-Eleni Mastrodima, Efstathios Zarkadoulas, Dimitrios S. Kanakoglou, Dimitris Kekos, Michalis Pachnos, George Mavridis, Diomi Mamma, Dimitris G. Hatzinikolaou

**Affiliations:** 1 Enzyme and Microbial Biotechnology Unit, Department of Biology, National and Kapodistrian University of Athens, Zografou Campus, 15784 Athens, Greece; 2 Department of Biological Chemistry, Medical School, National and Kapodistrian University of Athens, 75 Mikras Asias Street, 11527 Athens, Greece; 3 Biotechnology Laboratory, Sector of Synthesis and Development of Industrial Processes (IV), School of Chemical Engineering, National Technical University of Athens, Athens, Greece; 4 Division of European Affairs, Motor Oil Hellas, 15121 Marousi, Athens, Greece

**Keywords:** Biodesulfurization, dibenzothiophene, biphasic system, diesel oil, biocatalyst stability

## Abstract

Sustainable biodesulfurization (BDS) processes require the use of microbial biocatalysts that display high activity against the recalcitrant heterocyclic sulfur compounds and can simultaneously withstand the harsh conditions of contact with petroleum products, inherent to any industrial biphasic BDS system. In this framework, the functional microbial BDS-related diversity in a naturally oil-exposed ecosystem, was examined through a 4,6-dimethyl-dibenzothiophene based enrichment process. Two new *Rhodococcus* sp. strains were isolated, which during a medium optimization process revealed a significantly enhanced BDS activity profile when compared to the model strain *R. qingshengii* IGTS8. In biocatalyst stability studies conducted in biphasic mode using partially hydrodesulfurized diesel under various process conditions, the new strains also presented an enhanced stability phenotype. In these studies, it was also demonstrated for all strains, that the BDS activity losses were decoupled from the overall cells' viability, in addition to the fact that the use of whole-broth biocatalyst positively affected BDS performance.

## 1. Introduction

Combustion of crude oil-derived fossil fuels that contain sulfur compounds leads to the release of sulfur oxides into the atmosphere, increasing air pollution and contributing to the deterioration of environmental quality [1]. Environmental protection agencies worldwide have imposed strict regulations on combustible fossil fuel products. The current desulfurization technology of oil products involves the catalytic reduction of organic sulfur to H_2_S at elevated temperatures using hydrogen through the process of hydrodesulfurization (HDS). While the process works well for thiols and sulfides, it becomes energy-intensive when it reaches the temperatures required to remove heterocyclic sulfur compounds (thiophene derivatives). This step is necessary for achieving the high desulfurization levels imposed by regulations [2]. In addition to the elevated cost, these temperatures also result in quality deterioration of the final product. As a result, alternative technologies are being explored, mainly targeting petroleum products' recalcitrant heterocyclic sulfur compounds [2],[3]. Among them, the most promising is the biodesulfurization (BDS) process. This term denotes the microbially mediated removal of benzothiophene (BT), dibenzothiophene (DBT), and their alkylated homologs from various oil products when in contact with microbial biomass (biphasic systems). The process has been reported to be associated with diverse microbial genera and can be potentially used for industrial-scale desulfurization [4].

**Figure 1.**
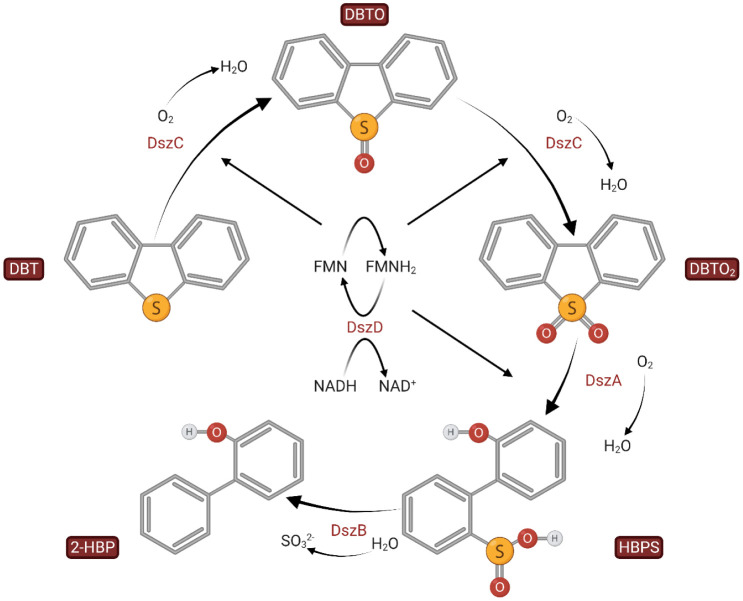
The 4S pathway for the biocatalytic desulfurization of DBT and its derivatives. Final editing was concluded with the tools provided by BioRender.com.

In literature, most BDS studies have focused on DBT as the model heterocyclic sulfur compound. This fact has resulted in the explication of two major biochemical utilization pathways, the ring destructive (degradation) or “Kodama pathway” and the sulfur-specific (desulfurization) "4S" sulfur removal pathway. The Kodama pathway is often characterized as a "destructive" BDS pathway, in which carbon-carbon bonds in the DBT molecules are broken, leading to the loss of only one phenolic ring with the S molecule remaining in an organic form. As a result, the majority of research has been focused on the 4S pathway (Figure 1), as it is an oxidative desulfurization pathway that cleaves the carbon-sulfur bond in DBT, yielding 2-hyrdoxybiphenyl (2-HBP) and sulfite ions as final products, thus not affecting the carbon content of the processed fuel [5]. Few researchers have further demonstrated the existence of an extended 4S pathway in some bacterial species such as *Achromobacter* and *Mycobacterium* spp, where the 2-HBP formed from DBT is partially converted to 2-methoxybiphenyl (2-MBP), although the exact mechanism and enzymes involved in this conversion remain uncharacterized [6]–[8]. The first characterized desulfurization pathway is from *Rhodococcus qingshengii* strain IGTS8 [9], formerly identified as *R. erythropolis* [10]. It involves the enzymes that catalyze the cleavage of carbon-sulfur bond and the well-characterized genes that encode them. The desulfurization operon designated as *dszABC* lies in a 150 kb plasmid and includes three genes that encode enzymes for the progressive conversion of DBT to 2-HBP. The reductive power required is supplied through the action of an NADH-FMN oxidoreductase, namely DszD, encoded in the genomic DNA. The conversion process comprises of three steps. During the first two steps, DBT is converted to dibenzothiophene sulfoxide (DBTO) and subsequently to dibenzothiophene sulfone (DBTO_2_). The third step involves the conversion of DBTO_2_ to 2-hydroxybiphenyl-2-sulfinate (HBPS) and ultimately to 2-HBP (Figure 1) [2]. This *dsz* genotype appears to be well preserved among a significant number of diverse genera that share homologs of Dsz enzymes [11],[12].

Over the last twenty years, a significant amount of research work has been conducted on various aspects of the BDS process, including the quest for new and more efficient wild-type biodesulfurizing strains, the optimization of their culture conditions and performance in various BDS-reactor configurations, the homologous or heterologous overexpression of the Dsz enzymes into miscellaneous hosts etc. [13]–[15]. Despite this, BDS is still far from any economically viable industrial application. Several reasons have been stated for this situation, with the most prominent being the low overall biodesulfurizing activity of the microbial biocatalysts, that is usually accompanied by their low viability and stability, especially when in contact with petroleum products [16].

Targeting towards the latter two important impediments for enhanced BDS in this work, the bioprospecting for new microbial isolates with enhanced BDS phenotypes was pursued. For strain isolation a unique Greek environment was selected, namely the Keri marsh in the island of Zakynthos, Western Greece, an area characterized by slow and continuous crude oil escapes from the ground. Considering the complexity of heterocyclic sulfur sources that are present in petroleum products, and the increased recalcitrancy of the alkylated forms of DBT that are the primary S-compounds in diesel [17],[18], an enrichment process of the soil samples based on 4,6 dimethyl dibenzothiophene (4,6-DM-DBT) was performed aiming to identify more robust BDS biocatalysts [19],[20]. Following the isolation of new biodesulfurization strains and the evaluation of their performance as BDS biocatalysts, we additionally developed an elaborate stability-viability assay in an effort to examine the stability and viability kinetics of both cells and related BDS activity in biphasic systems using model and real-life diesel samples.

## 2. Materials and methods

### 2.1. Study site and sampling campaign

The sampling area (supplementary data, Figure S1) is Keri Lake's ancient so-called 'Herodotus Springs'. It is a coastal ecosystem in the southern part of Zakynthos Island, in the Ionian Sea. Its area is approximately 30,000 m^2^ with an average elevation of 1 m above sea level [21]. Keri Lake is separated from the open sea by a low sand barrier, limiting communication with the open sea. The initial limnic environment evolved into a coastal fen covered mainly with reeds, which led to the accumulation of a 5 m thick peat bed below the fen environment [21]. The oil escape at Keri Lake has been a well-known physical phenomenon for 2500 years (Herodotus, Book IV, Chapter 195). Former geochemical analyses of the escaping oil have revealed a marine organic matter origin with a high maturity level and abundance of aromatic compounds [22]. Five crude-oil contaminated sediment samples were collected randomly from the sampling area. Sediment samples (10 cm deep) were taken under aseptic conditions, using sterile equipment. The samples were transferred into 50 mL polypropylene Falcon tubes using sterilized spatulas, preventing air exposure as much as possible. The tubes were stored at 4–6 °C and transferred to the lab within 24 hours where they were kept at the same temperature (for a maximum of one week) until further processing.

### 2.2. Isolation media

The sulfur-free chemically defined medium (CDM) was used throughout this work. It was comprised of (in g/L): NaH_2_PO_4_·H_2_O, 3.8; Na_2_HPO_4_·7H_2_O, 3.3; NH_4_Cl, 0.8; NaCl, 8.5; KCl, 0.5. The following components were added to the autoclaved medium, from filter-sterile stock solutions: Solution C: (1 mL/L)-MgCl_2_·6H_2_O (324 g/L) and CaCl_2_·2H_2_O (29.4 g/L), Solution D: (10 mL/L)-containing (per L) Na_2_SeO_3_·5H_2_O, 6 mg; Na_2_WO_4_·2H_2_O, 8 mg; and NaOH, 0.4 g. Trace element stock solution: (1 mL/L)–Na_2_·EDTA (5.2 g/L); FeCl_2_·4H_2_O (3 mg/L); H_3_BO_3_ (30 mg/L); MnCl_2_·4H_2_O (100 mg/L); CoCl_2_·6H_2_O (190 mg/L); NiCl_2_·6H_2_O (24 mg/L); CuCl_2_ (0.2 mg/L); ZnCl_2_ (0.5 mg/L); and Na_2_MoO_4_·2H_2_O (36 mg/L). Vitamin stock solution (1 mL/L), containing (per L): D (+) biotin, 10 mg; 4-aminobenzoic acid, 40 mg; nicotinic acid, 100 mg; calcium D-(+)-pantothenate, 50 mg; pyridoxamine-dihydrochloride, 25 mg; thiamin dihydrochloride/L, 50 mg; vitamin B12, 50 mg. This medium was supplemented, depending on the experiment, with different carbon (glucose, glycerol, ethanol) and sulfur (dimethyl sulfoxide-DMSO, dimethyl sulfone-DMSO_2_, taurine, DBT, 4,6-DM-DBT, sodium sulphate) sources at the indicated concentrations. In the experiment for the nitrogen source effect, NH_4_Cl was replaced with other nitrogen sources (NH_4_NO_3,_ NaNO_3_, urea). When used, DBT was added from 100 mM ethanol stock while 4,6-DM-DBT was similarly prepared in acetone. All media were prepared in high purity (HPLC grade) water (PanReac, Applichem). A slight adjustment with 1M NaOH was performed, when necessary, in order to reach an initial value of pH 7.

### 2.3. Enrichment cultures and isolation of bacteria

Enrichment cultures under aerobic conditions were performed in CDM with 0.5 mM 4,6-DM-DBT as sulfur source and 110 mM glycerol as carbon source and incubated at 30 °C under shaking (180 rpm). Erlenmeyer flasks (100 mL) were filled with 20 mL medium and inoculated with 2 grams (wet weight) of the sediment sample. The incubation lasted for seven days when 2 mL of the respective enrichment culture was transferred into 18 mL fresh medium which was further incubated for additional seven days. Following five enrichment cycles, whole culture broth from the enrichment cultures were serially diluted in sterile PBS. 0.1 mL from the 10^−2^, 10^−4^, and 10^−6^ serial dilutions were spread on the same enrichment medium agar plates. The plates were prepared in duplicate and incubated at 30 °C. The CDM plates were incubated for 72 h, and the developed colonies were purified by repeated streaking on the same medium till pure colonies were obtained.

### 2.4. Molecular characterization of the isolates

Bacterial isolation and purification of genomic DNA were performed using the NucleoSpin Microbial DNA purification kit (Macherey-Nagel), according to the manufacturer's recommendation. DNA quantification was determined using Thermo Scientific NanoDropTM 2000c Spectrophotometer. Strain identification was based on the amplification of the 16S rRNA gene using the universal primers 27F and 1492R, which span nearly the entire length of the gene with an expected amplicon of ~1400bp (supplementary data, Table S1). The PCR products were directly sequenced using a BigDye terminator cycle sequencing. The nucleotide sequences were then submitted to GenBank (accession numbers ON678184 to ON678199 and ON680692).

To confirm the existence of desulfurization (*dsz*) genes in the isolates' genomes, primers based on known sequences from *R. qingshengii* IGTS8 (GenBank accession numbers U08850.1 for *dszABC,* CP029297.1 for *dszD*) were used (supplementary data, Table S1). All PCR amplifications were performed using Taq DNA polymerase (MBI Fermentas, Lab Supplies, Athens, Greece), according to the manufacturer's instructions. DNA and amino acid sequences were analyzed using the BLAST programs of the National Center for Biotechnology Information.

### 2.5. Phylogenetic characterization by maximum likelihood method

The evolutionary history was determined through the Maximum Likelihood (ML) method and Tamura-Nei model [23]. The tree with the highest log likelihood (-38750.95) is shown. Initial tree for the heuristic search was obtained automatically by applying Neighbor-Join (NJ) and BioNJ [24] algorithms to a matrix of pairwise distances estimated using the Tamura-Nei model, and then selecting the topology with superior log likelihood value. The final dataset comprised of 54 partial 16S ribosomal RNA (16S rRNA) gene sequences retrieved from GenBank. The phylogenetic tree was constructed using partial 16S rRNA gene sequences. Alignment and nucleotide content was calculated using the general-purpose nucleotide search and alignment program BLASTn. Furthermore, MUSCLE was used for aligning the nucleotide sequences. Evolutionary analyses were conducted in MEGA11 [25]. The Newick notation was then transferred to the online tool iTOL (Interactive Tree Of Life) [26] for further annotation and data implementation.

### 2.6. Physiological characterization of the biodesulfurization phenotype

To examine the effect of different media on growth and desulfurization activity, the wild-type strain *R. qingshengii* IGTS8 and the isolated strains *R. qingshengii* ATHUBA4003 and ATHUBA4006 were grown in CDM pH = 7.0, under different carbon, nitrogen, and sulfur sources of a predefined concentration. Growth took place in 96-well cell culture plates (F-bottom; Greiner Bio-One, Fischer Scientific, US) in thermostated plate shakers at 30 °C and 600 rpm as previously described [27]. An initial biomass concentration of 0.045–0.055 g/L (dry cell weight) was universally applied, while 60 identical well-cultures were used per condition (150 µL culture per cell). Biomass concentration, expressed as Dry Cell Weight (DCW), was estimated by measuring the optical density at 600 nm with a Multiskan GO Microplate Spectrophotometer (Thermo Fisher Scientific, Waltham, MA USA), using an appropriate calibration curve.

Desulfurization activity was determined through a resting cells assay [28]. In brief, the content of 4 to 8 wells from each condition (depending on the growth stage) was removed, mixed in a 1.5 mL Eppendorf tube, centrifuged for 5 min at 1000xg, and cell pellets were resuspended in 50 mM Hepes buffer (pH = 8.0) to a final concentration between 0.5 and 2 g DCW/L. One hundred and fifty µL of cells' suspension were added to 150 µL of 2 mM DBT in the same buffer, in an Eppendorf tube. The tube was incubated in a thermoshaker at 1200 rpm and 30 °C for 30 min. Then, 300 µL acetonitrile was added under stirring, and the tubes were centrifuged to remove the cells. A tube with equivalent amounts of cells and acetonitrile without incubation (t = 0) was used as blank. The supernatant was assayed for 2-HBP determination by HPLC with fluorescence detection [27]. BDS activity was expressed as Units per mg DCW, with 1 U corresponding to the release of 1 nmole 2-HBP per h under the above-described conditions. Final biomass concentration in the collected cells' suspension between 0.5 and 2 g DCW/L did not affect resting cell BDS results (Units/mg DCW) for *R. qingshengii* IGTS8, ATHUBA4003 or ATHUBA 4006, based on One-way ANOVA with Tukey's multiple-comparison test (confidence interval 95%; n = 2).

### 2.7. Stability studies on biphasic BDS systems

Biphasic model systems were prepared at three different volumetric organic-to-aqueous (OA) ratios, 40/60, 60/40, and 80/20, at a total volume of 1.2 mL in 2 mL Eppendorf tubes. In all experiments, the organic phase comprised partially desulfurized (through HDS) diesel fuel with nominal total sulfur content equal to 100 ppm (obtained from Motor-Oil S.A. refinery at Agioi Theodoroi, Korinthos, Greece). For the aqueous phases, *R. qingshengii* IGTS8, ATHUBA4003, and ATHUBA4006 strains were grown in CDM with 165 mM ethanol, 1.3 mM DMSO, and 15 mM NH_4_Cl, as carbon, sulfur, and nitrogen sources, respectively. Growth took place in 250 mL conical flasks (50 mL working volume, 200 rpm) and was closely followed by OD_600_ measurements up to a cell concentration of 0.5 ± 0.05 mg DCW/mL (approx. mid-exponential phase for all strains). For biphasic BDS system preparation two types of aqueous phases were prepared: In the first, the culture broth was centrifuged, the supernatant discarded, and the cells were washed two times with Ringer solution and resuspended into 50 mM HEPES buffer pH = 8 at a final biomass concentration of 0.5 ± 0.05 mg DCW/mL. Whole culture broth of the same biomass concentration was used directly as the second type of aqueous phase examined. Six biphasic tubes were prepared per condition as described above (12 conditions in total) and were placed in an orbital shaker operating at 1200 rpm and 30 °C. After 4 and 24 hours, three identical tubes were removed from each condition, the upper-organic phase was carefully aspirated, and the lower aqueous phase was used for the determination of the remaining BDS activity (as described above) of the cells, as well as the viable-cell counts, through serial dilutions and spreading on Nutrient-Agar plates.

## 3. Results and discussion

### 3.1. Screening for 4,6-dimethyl-DBT-desulfurizing isolates

**Figure 2.**
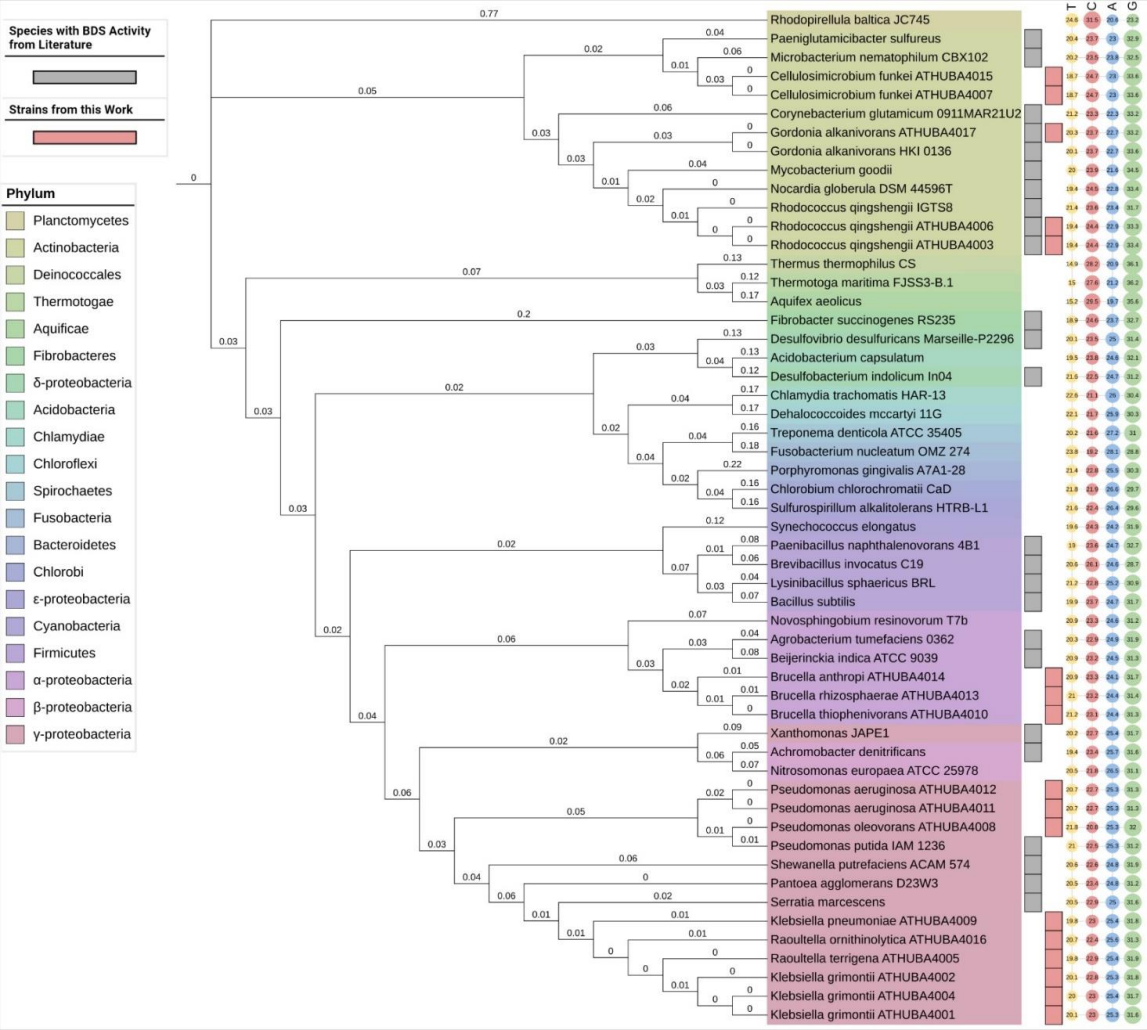
Maximum Likelihood (ML) phylogenetic tree of the isolates from Keri marsh in Zakynthos island, Greece.

The performed enrichment procedure resulted in a microbial consortium that grew in a chemically defined medium containing glycerol and 4,6-DM-DBT as sole carbon and sulfur source, respectively (see Materials and methods). Serial dilutions of the final enrichment subculture plated on the same solid culture medium (on glycerol and 4,6-DM-DBT) revealed several morphologically distinct colonies indicating a mixed culture. A total of 16 distinct strains were isolated and assigned in seven different genera according to their 16S rRNA sequences (Table 1). Furthermore, in order to highlight the phylogenetic position of the isolated strains relative to *Rhodococcus qingshengii* IGTS8 and other desulfurizing strains from the literature, a phylogenetic tree (Figure 2) was constructed using partial 16S ribosomal RNA (16S rRNA) sequences. The final dataset comprised 54 partial 16S rRNA gene sequences retrieved from GenBank.

As inferred from Figure 2, the isolated strains cover a wide range of bacterial classes, from actinobacteria to γ-proteobacteria. Among them are included well-studied desulfurizing species belonging to *Rhodococcus* [3],[29],[30] and *Gordonia* [31] genera, as well as genera never reported before for the desulfurization of heterocyclic organosulfur compounds like *Brucella*, *Raoultella* and *Cellulosimicrobium* spp. Finally, wild-type strains of the *Klebsiella* and *Pseudomonas* genera, that are found in the KL consortium have also been scarcely used in BDS studies [12] or identified as members of desulfurizing microbial communities [32],[33].

**Table 1.** Identification of bacterial strains by 16S rRNA analysis.

Isolated strains in this work		16s rRNA identification [% identity to the type strain]		
Strain name	GenBank accession no.	Type Strains	% Identity	GenBank accession no.
*Klebsiella grimontii* ATHUBA4001	ON678184	*Klebsiella grimontii* strain SB73	99.78	NR_159317.1
*Klebsiella grimontii* ATHUBA4002	ON678185	*Klebsiella grimontii* strain SB73	99.78	NR_159317.1
*Rhodococcus qingshengii* ATHUBA4003	ON678186	*Rhodococcus qingshengii* strain CCM 4446	100	NR_115708.1
*Klebsiella grimontii* ATHUBA4004	ON678187	*Klebsiella grimontii* strain SB73	99.86	NR_159317.1
*Raoultella terrigena* ATHUBA4005	ON678188	*Raoultella terrigena* strain 84	99.78	NR_037085.1
*Rhodococcus qingshengii* ATHUBA4006	ON678189	*Rhodococcus qingshengii* strain JCM 15477	100	NR_043535.1
*Cellulosimicrobium funkei* ATHUBA4007	ON678190	*Cellulosimicrobium funkei* strain W6122	99.78	NR_042937.1
*Pseudomonas oleovorans* ATHUBA4008	ON678191	*Pseudomonas oleovorans* strain RS1	99.93	NR_115874.1
*Klebsiella pneumoniae* ATHUBA4009	ON678192	*Klebsiella pneumoniae* strain R-70	100	NR_037084.1
*Brucella thiophenivorans* ATHUBA4010	ON678193	*Brucella thiophenivorans* strain DSM 7216	99.38	NR_042599.1
*Pseudomonas aeruginosa* ATHUBA4011	ON678194	*Pseudomonas aeruginosa* strain DSM 50071	100	NR_117678.1
*Pseudomonas aeruginosa* ATHUBA4012	ON678195	*Pseudomonas aeruginosa* strain DSM 50071	100	NR_117678.1
*Brucella rhizosphaerae* ATHUBA4013	ON678196	*Brucella rhizosphaerae* strain PR17	99.54	NR_042600.1
*Brucella anthropi* ATHUBA4014	ON678197	*Brucella anthropi* strain LMG 3331	100	NR_114979.1
*Cellulosimicrobium funkei* ATHUBA4015	ON678198	*Cellulosimicrobium* funkei strain W6122	99.70	NR_042937.1
*Raoultella ornithinolytica* ATHUBA4016	ON678199	*Raoultella ornithinolytica* strain CIP 103364	99.93	NR_044799.1
*Gordonia alkanivorans* ATHUBA4017	ON680692	*Gordonia alkanivorans* strain HKI 0136	99.93	NR_026488.1

The fact that all isolated microorganisms originate from an enrichment culture containing 4,6-DM-DBT as a sole sulfur source does not guarantee that they metabolize DBT and its alkylated derivatives without attacking their hydrocarbon skeleton, i.e., through the 4S metabolic pathway (Figure 1). This was true also in the present study, wherein all strains were able to grow on CDM with DBT as the sole carbon source, but significant amounts of 2-HBP–the end-product of the 4S-pathway–were detected only in the *R. qingshengii* ATHUBA4003 and 4006 cultures (data not shown). The same result was obtained in resting cells assays performed by cells grown on CDM with DMSO as the sulfur source, where 2-HBP was detected as the final product only in the two isolates mentioned above. These results inferred that only the two isolated *Rhodococcus* strains are metabolizing DBT through the 4S-pathway. The remaining isolates that could grow on and presumably assimilate DBT and 4,6-DM-DBT without producing the corresponding 4S-related end-products most probably utilize a different pathway that does not end up in 2-HBP formation [34],[35]. One known alternative is the Kodama pathway that proceeds by retaining the C-S bond [36] and has been demonstrated to function in *Pseudomonads* and other proteobacteria [37],[38]. Even for *Gordonia* spp, a genus with well-known 4S desulfurizing members, additional desulfurizing pathways might exist since, upon growth on benzothiophene, the upregulation of several additional non-4S related genes has been verified [39].

Following these results, our work was subsequently focused in evaluating the biodesulfurization performance of the two novel *R. qingshengii* isolates, ATHUBA 4003 and 4006, in reference to the model *R. qingshengii* IGTS8 strain.

### 3.2. Biodesulfurization activity in resting cells' assays

The resting cells' assay has been extensively used in literature to evaluate the desulfurization potential of bacterial cells as a function of different growth conditions [40]. It consists of adding a certain amount of biomass in a buffered solution of DBT and measuring the amount of 2-HBP produced, following incubation for a certain amount of time, under stirring. Although performed in aqueous conditions, it is the most precise way to estimate the overall Dsz enzymes' activity, at the time point of cell harvesting from the growing culture.

#### 3.2.1. Effect of selected carbon sources on growth and desulfurization activity

**Figure 3.**
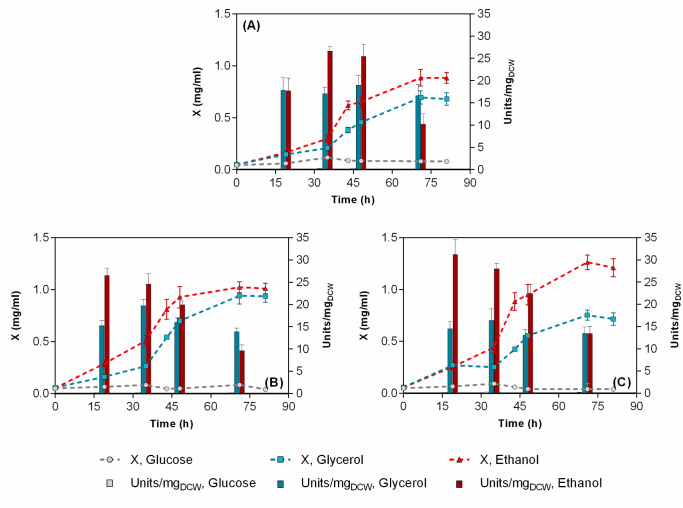
Growth curves (line-symbol plots) and specific desulfurization activities (bar plots) during growth of (A) *R. qingshengii* IGTS8, (B) *R. qingshengii* ATHUBA4003, and (C) *R. qingshengii* ATHUBA4006 in CDM supplemented with different carbon sources (at 330 mM-C). NH_4_Cl at 15 mM, and DMSO at 1.3 mM was used as nitrogen and sulfur sources, respectively. Data for glycerol and glucose in (Α) are from (Martzoukou et al. 2022). “X” represents biomass concentration (mg dry cell weight /mL).

Carbon source is a substantial cost factor in any biotechnological process and will affect the economic viability of any potentially commercialized BDS process [40],[41]. Thus, we initially examined the effect of carbon source type (glucose, glycerol, and ethanol at 0.33 M carbon) in a low-cost simple salts' medium like CDM, with DMSO as a sole sulfur source (see Section 2). DMSO was selected instead of DBT since it does not have a repressive effect on the *dsz*-operon [42], in addition to the fact that it is a more convenient sulfur source than DBT for the production of industrial amounts of biocatalyst because of its cost and availability. Finally, at the applied concentration (1.3 mM) it is soluble in water and does not interfere with OD measurements (in contrast to DBT). Figure 3 and related supplementary data Table S2 present the corresponding results.

Among the carbon sources tested, ethanol proved to be the most efficient in terms of growth and desulfurization efficiency. In this carbon source, strain ATHUBA4006 grew slightly better compared to the other two strains reaching a maximum biomass concentration at the levels of 1.3 g DCW/L. Glycerol supported statistically significant lower growth levels, with ATHUBA4003 isolate showing the highest maximum biomass concentration. Similar maximum BDS activity values were obtained for all strains during the early to mid-log phase (~ 25–30 U/mg_DCW_). In glycerol, BDS-specific activity was much less affected by the growth phase, with a maximum value not exceeding 20 U/mg_DCW_ for all three strains. It is noteworthy that under the medium matrix employed and the specific experimental conditions, glucose could not establish sufficient growth and any associated BDS activity for any strains.

Several studies have addressed the selection of carbon source type in relation to BDS activity in rhodococci [40],[43]. Glycerol is frequently used in BDS studies without justification, probably due to its low cost and availability, either supplementing a simple salt medium [11],[44] or in combination with complex nitrogen sources such as yeast extract [45]. Ethanol is less frequently used, although it has been shown to be the best among the examined carbon sources in fed-batch cultures of *R. erythropolis* KA-2-5-1 when it was simultaneously fed with DBT [43],[46] probably due to the additional reducing power (NADH) that provides during its catabolism, a fact that has also been predicted through flux-based analysis of sulfur metabolism in *R. erythropolis* [47]. In the present work, this seems to be also the case when ethanol is used for BDS catalyst production in combination with DMSO as the sole sulfur source. The inability of glucose to support the growth of all three *R. qingshengii* strains when supplied in a minimal salt medium with DMSO as sole sulfur source is a novel and rather unexpected result. Having in mind that in *Gordonia alkanivorans* [48] glucose can induce a lag-phase of more than 72 h, the glucose cultures were incubated for more than a week without achieving any substantial growth level, similarly to those depicted in Figure 3. A more scrutinous look on the relevant studies reveals that glucose had been used as carbon source in the past, but until recently, never in a minimal salt medium in combination with DMSO as the sole sulfur source [27]. Even in *R. qingshengii* IGTS8 studies where glucose (at 20 g/L) and DMSO are claimed to be the best combination of carbon and sulfur sources, respectively, with respect to growth and BDS activity, the basal medium employed contained an additional 20 g/L of glycerol [29],[40].

#### 3.2.2. Effect of selected sulfur sources on growth and desulfurization activity

**Figure 4.**
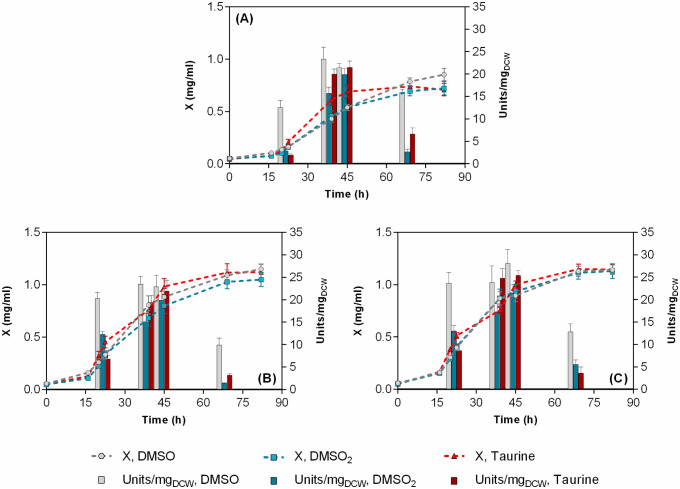
Effect of sulfur source type (at 1.3 mM), on growth (line-symbol plots) and specific desulfurization activities (bar plots) of (A), *R. qingshengii* IGTS8; (B), *R. qingshengii* ATHUBA4003; and (C), *R. qingshengii* ATHUBA4006 in CDM. Ethanol (165 mM) and NH_4_Cl at 0.8 g/L (15 mM) were used as carbon and nitrogen sources, respectively. “X” represents biomass concentration (mg/mL).

In order to study the influence of sulfur source type, the growth of the microorganism using different types of water-soluble sulfur sources, namely, DMSO, DMSO_2,_ taurine, and sulphate ions (as Na_2_SO_4_), has been carried out at an initial concentration of 1.3 mM, using ethanol as a carbon source (Figure 4). Τhere were no statistically significant differences in the growth pattern of each strain, with respect to the sulfur source used. As in the previous experimental set, *R. qingshengii* ATHUBA4003 and 4006 presented higher overall growth rates and almost 40% higher biomass yields compared to *R. qingshengii* IGTS8. Sulphate ions, although supported equivalent growth with the other S-sources, did not induce any significant BDS activity within the cells (data not shown). As far as the other three sulfur sources are concerned, DMSO was marginally the best inducer/non-repressor of BDS activity, with the positive effect being more profound at the beginning of the exponential and the stationary phases. Maximum BDS activity of the two new isolates, ATHUBA4003 and ATHUBA4006, was slightly higher than that of IGTS8 but the difference was statistically insignificant. When this result though, is combined with the higher biomass yield mentioned above, yields in an average 50% and 100% higher BDS volumetric productivity (Units/L culture volume) for ATHUBA4003 and ATHUBA 4006, respectively (supplementary data, Table S2).

Only a few studies have systematically addressed the effect of various S-sources on BDS activity, while in most of them there is no clear justification on the choice of the S-source used. This work is focused on the examination of non *dsz*-operon-repressing, water-soluble, and low-cost sulfur sources, like DMSO, DMSO_2_, taurine and sulphate. As expected, SO_4_^−^ ions at the applied concentration almost completely repressed the expression of the Dsz enzymes and the corresponding BDS activity. It is well-known that the BDS phenotype is repressed upon sulphate addition and relatively recently has been shown that this effect is probably due to the increase in the intracellular cysteine pool–which has an important role in the repression of the *dsz*-operon-caused by excess sulphate [42],[49]. DMSO has been often reported as the best sulfur source since it does not suppress the expression of the *dsz*-operon promoter [50]. Its use as the sole sulfur source to produce BDS biocatalysts is thus very often mentioned in literature and provides for enhanced specific BDS activity-even better than DBT–in addition to promoting better growth [40],[51]. Since DBT is immiscible with water, hence unsuitable for use in a practical commercial process, these results supported the notion that DMSO can be used as a water-soluble sulfur source for cultivation of *Rhodococcus* strains without causing an inhibitory effect on growth and hence higher desulfurization productivities can be achieved.

#### 3.2.3. Nitrogen source effect on growth and desulfurization activity

**Figure 5.**
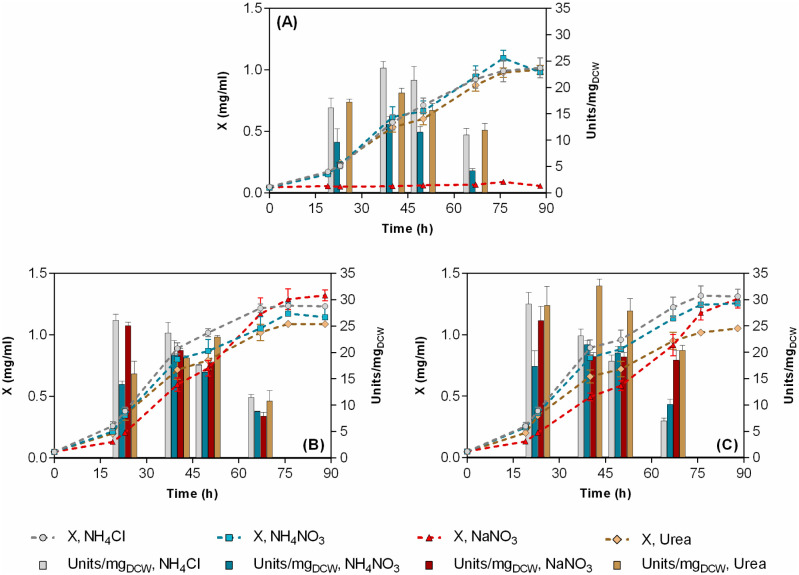
Effect of nitrogen source type (at 15 mM-N), on growth (line-symbol plots) and specific desulfurization activities (bar plots) of (A) *R. qingshengii* IGTS8, (B) *R. qingshengii* ATHUBA4003, and (C) *R. qingshengii* ATHUBA4006 in CDM. Ethanol (165 mM) and DMSO (1.3 mM) were used as carbon and sulfur sources, respectively. “X” represents biomass concentration (mg/mL).

Finally, the effect of nitrogen source type on growth and BDS activity of cells grown in cultures with ethanol and DMSO as carbon and sulfur sources, respectively, was examined (Figure 5, supplementary data Table S4). As in the two previous experimental sets, the two new *R. qingshengii* isolates performed significantly better than the model IGTS8 strain, both in terms of growth and specific BDS activity. One notable observation is that, in contrast to *R. qingshengii* ATHUBA4003 and 4006 isolates, *R. qingshengii* IGTS8 failed to grow in the medium with NaNO_3_ as the sole nitrogen source. An extensive literature search identified a limited number of studies not related to BDS, where *Rhodococcus* spp. were grown in only NO_3_^−^ as nitrogen source [52],[53]; however, the growth of *R. qingshengii* IGTS8 on this nitrogen source has not been assessed previously. The recent genome announcement for IGTS8 identified certain nitrate-reducing enzymes and a nitrate/nitrite transporter (IGTS8_peg957, IGTS8_peg759, IGTS8_peg760, IGTS8_peg956, IGTS8_peg62) [10] and this is also the case for other *Rhodococcus* isolates [54]. The capability of the two new strains to grow on nitrate probably reflects their environmental origin, a hypothesis supported by the experimental results, specifically from the Keri marsh, in which nitrate levels in oil-impacted sites appeared to be enhanced compared to the non-oil-impacted ones [55]. This observation probably explains the more efficient uptake and/or catabolism of nitrate as the sole nitrogen source by *R. qingshengii* ATHUBA4003 and ATHUBA4006, although with significantly lower rates compared to the other sources.

As far as the other three nitrogen sources examined, ammonium chloride and urea supported higher BDS activities for all strains, although NH_4_Cl was significantly better in supporting microorganisms' growth. This goes in line with the majority of biodesulfurization studies where ammonium chloride is the preferred nitrogen source used for biocatalyst production [13],[40],[51]. It is widely known that most microbes preferentially utilize ammonium in order to avoid having to accomplish nitrate reduction-an energy-requiring process, but also, to avoid the inhibitory effects due to the decrease in the water activity and the extreme toxicity of nitrite and nitric oxide produced during the first steps of nitrate reduction to ammonium [56]. Finally, urea, a low-cost nitrogen source, has never been used before for BDS-biocatalyst production, but in the present work has been proven equally suited for enhanced specific BDS activity. It is quite interesting that for *R. qingshengii* ATHUBA 4003 and 4006, the maximum specific BDS activity with urea as nitrogen source, was observed at mid-log culture stages compared to the ammonium cultures where maximum BDS was achieved at the early log-phase. Combined with the lower growth kinetics in urea cultures, this observation probably reflects the time needed to reach the optimum expression levels of urease, known to be present in *Rhodococcus* genome [10].

### 3.3. Stability in biphasic BDS systems

**Figure 6.**
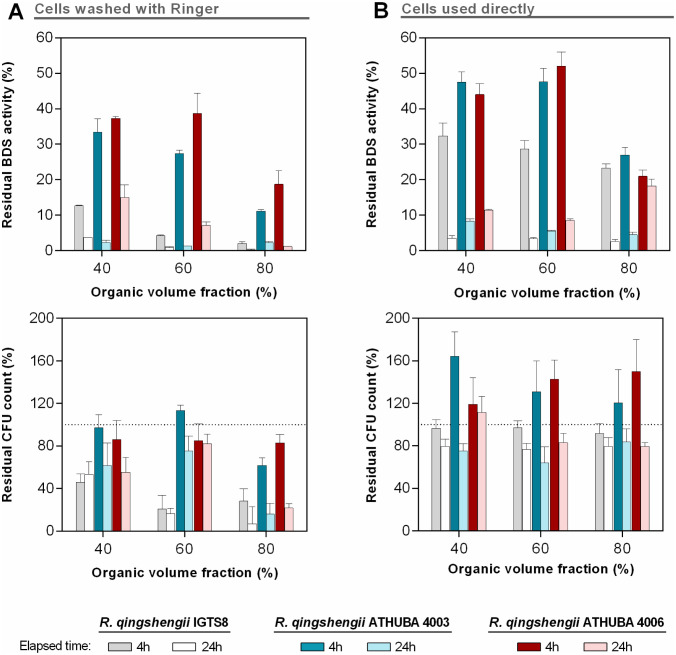
Residual BDS activity (upper panels) and cell viability (CFU count) (lower panels) of the microbial biomass in a biphasic system with diesel fuel. Cells were removed from cultures, at the optimum conditions (ethanol, DMSO, NH_4_Cl), washed twice with Ringer's solution (A) or used directly (B) in the biphasic system, at a concentration of 0.5 ± 0.05 mg_DCW_/mL in the aqueous phase. For all three strains, the initial (t = 0 h) BDS activity was between 19.4 and 24.2 U/mg DCW and the initial CFU count between 6.9 and 8.7 x 10^8^ CFU/mL.

In addition to a high biodesulfurizing ability, a candidate biocatalyst should be able to withstand the harsh environment of an industrial-scale biphasic BDS system. To test the inherent stability of the new strains when in contact with petroleum products, we performed a series of lab-scale biphasic experiments using an HDS desulfurized diesel where both the viability of the added biocatalyst as well as its remaining BDS activity, at different oil-to-aqueous (O/A) phase ratios and different contact times were examined. The O/A phase ratios employed were 40%, 60% and 80% v/v, since lower ratios have no practical use in industrial BDS economics (Haruna et al. 2022; Acero, Berdugo, and Mogollón 2003). Two types of aqueous phases were additionally evaluated: In the first (Figure 6A), cells were washed and resuspended in HEPES buffer at the required concentration, while in the second (Figure 6B), whole broth at the same concentration was used directly as the aqueous phase (see Materials and Methods).

The contact of the aqueous phase with the HDS diesel in a biphasic biodesulfurization environment has resulted in significant losses of the BDS activity especially for the washed biocatalyst cells (Figure 6A-left diagrams). In the case of *R. qingshengii* IGTS8 the activity loss is very significant reaching 85 and 96% after 4 and 24 h incubation at the 40/60 oil-to-aqueous (O/A) ratio. At higher O/A ratios the losses were even greater. The stability profile for the two new *R. qingshengii* strains were significantly better though, especially at the 4 h incubation time, probably due to the prolonged exposure to crude oil in their natural habitat that transfused them with the necessary genetic traits to withstand diesel toxicity. It cannot be conferred at this stage though, whether this decreased rate of activity loss for the two new *R. qingshengii* strains, is a result of more stable 4S enzyme structures or a consequence of their evolutionary history that provides them with membranes less permeable to the toxic or enzyme inhibiting compounds of diesel.

Another very important observation is the fact that in general, the determined BDS activity losses are not directly related to the corresponding viability losses. This observation reflects the fact that during a biphasic BDS system, the 4S-pathway enzymes lose their functionality at a much greater rate compared to cells' survival mechanism. The two new strains though, lost on average only a small fraction of their viability (between 10 to 25%) upon 4 h contact with the diesel even at the high O/A ratio. In fact, high viabilities (over 50%) were maintained after 24 h incubation in the biphasic system for the two lower O/A ratios. The same pattern was also observed even for *R. qingshengii* IGTS8, but to a much lesser extent and basically only for the low O/A ratio of 40/60.

No studies where the biocatalyst's viability in a biphasic system with pragmatic (oil product) organic phases is correlated with the corresponding BDS activities were found, since such systems are usually studied through the kinetics of sulfur-content reduction as a function of various process parameters such as the O/A ratio, the biomass concentration in the aqueous phase etc. [57]. On the other hand, the most detailed studies on the quantitative aspects of a biphasic BDS system are performed using only “model-oils” where DBT and its derivatives are diluted in pure C12–C16 matrices [58],[59]. In a similar to the present work situation, but in a model-C16 system and 50/50 O/A ratio, *R. globerulus* resting cells lost almost completely their BDS activity after 24 h incubation while they maintained about 90% of their initial viability [60]. Finally in another very interesting study, with *Rhodococcus* sp. SA11 cells growing in a basal DBT-glycerol medium with the addition of different crude oil volumes up to a 50/50 ratio, the cells' viability was drastically decreased in the first 48 h compared to the culture with no oil addition [13]. In the same experiment, this viability difference was alleviated at higher growth times, but unfortunately no BDS activities were measured in parallel. The general trend though in this and other relevant studies is that when oil and oil-products O/A ratio is increased, a gradual reduction of the BDS conversion rates is observed, a fact that is also the case in the present work.

A second, equally important result with respect to the process economics was revealed when the use of the biocatalyst directly from the fermentation broth without any other treatment was examined, at equivalent conditions and concentrations in the biphasic systems (Figure 6B). A significant increase in both residual BDS activity and CFU count was observed for all three *Rhodococcus* strains especially at the 4h incubation time, in comparison with the corresponding use of washed biocatalyst. For the IGTS8 strain in particular, this increase was quite significant. Again, the two new isolates ATHUBA 4003 and 4006 revealed a better stability profile, retaining almost 50% of their initial BDS activity after 4 h in the 40/60 and 60/40 O/A ratios. These two isolates did not show any viability losses and, apparently, achieved some growth in the aqueous phases of all biphasic systems. The fact that this trend is observed for all three studied strains probably reflects either a protective effect of the spent growth medium on the intracellular physiology the Dsz-enzymes' activities or an opposite effect of the washing process (or a combination of both these factors).

In all referred resting-cells' biphasic studies with crude oil or oil derivatives as organic phase, the aqueous phase is comprised of cells grown in a Dsz-enzymes' inducing medium, collected at the time of maximum BDS activity, washed with a suitable buffer, and finally added in the biphasic system [13],[61]. It is easy to envisage that this final washing step is time- and cost-consuming when translated into a real-life industrial BDS system that requires large amounts of active biocatalyst. The fact that whole-broth-cells of the two new *R. qingshengii* isolates (and IGTS8) have revealed enhanced stability with respect to their BDS activity, compared to the washed cells in real-life biphasic biodesulfurization systems, is an overlooked characteristic of the overall biodesulfurization process cascade with obvious positive effect on process economics.

## 4. Conclusions

The targeted search of new biodesulfurization strains from environments of prolonged oil presence, can result in the isolation of microbial strains with improved BDS phenotypes. This was proven for the Keri marsh, the habitat from where two new *R. qingshengii* ATHUBA 4003 and ATHUBA 4006 were isolated. These strains have revealed elevated desulfurizing capabilities that where combined with enhanced stability properties as unveiled from a unique and diesel-oriented experimental approach that has been followed. Thus, they correspond to highly promising starting points for additional genetic and process optimization studies that could eventually lead to an economically viable industrial BDS process.
