# Supplementary material for: Deciphering the biodesulfurization potential of two novel Rhodococcus isolates from a unique Greek environment
Source: AIMS Microbiol. 2022 Dec 15;8(4):484–506. doi: 10.3934/microbiol.2022032 (PMC9834085; doi:10.3934/microbiol.2022032)
Supplement: Supplementary file 1 [file microbiol-08-04-032-s001.pdf]

Microbiology

AIMS Microbiology, 8(4): 484–506.
DOI: 10.3934/microbiol.2022032
Received: 09 September 2022
Revised: 10 November 2022
Accepted: 12 December 2022
Published: 15 December 2022

http://www.aimspress.com/journal/microbiology

Research article

Deciphering the biodesulfurization potential of two novel *Rhodococcus* isolates from a unique Greek environment

Panayiotis D. Glekas[1], Olga Martzoukou[1], Maria-Eleni Mastrodima[1], Efstathios Zarkadoulas[1], Dimitrios S. Kanakoglou[2], Dimitris Kekos[3], Michalis Pachnos[4], George Mavridis[4], Diomi Mamma[3,*] and Dimitris G. Hatzinikolaou[1,*]

[1] Enzyme and Microbial Biotechnology Unit, Department of Biology, National and Kapodistrian University of Athens, Zografou Campus, 15784 Athens, Greece
[2] Department of Biological Chemistry, Medical School, National and Kapodistrian University of Athens, 75 Mikras Asias Street, 11527 Athens, Greece
[3] Biotechnology Laboratory, Sector of Synthesis and Development of Industrial Processes (IV), School of Chemical Engineering, National Technical University of Athens, Athens, Greece
[4] Division of European Affairs, Motor Oil Hellas, 15121 Marousi, Athens, Greece

* **Correspondence:** to either: dhatzini@biol.uoa.gr; dmamma@chemeng.ntua.gr; Tel: +306932782004.

Supplementary

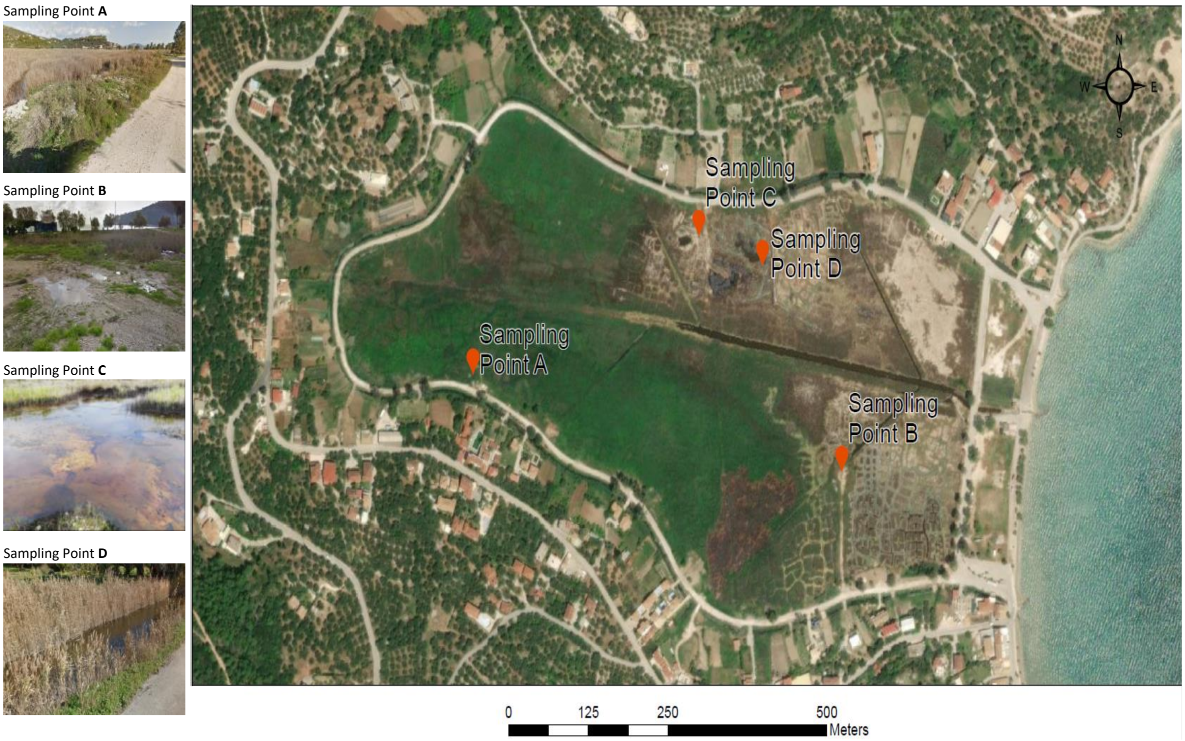

Figure S1. Map of Keri Lake showing the location of the sampling areas. Visualization was performed using Geographic Information Systems (GIS) technology.

Table S1 Oligonucleotides used in this study.

Primer name	Primer sequence (5'–3')	Target gene	Product size (bp)
dszA-F	ATGACTCAACAACGACAAATGCATCTG	*dszA*	1362
dszA-R	TCATGAAGGTTGTCCTTGCAGTTG		
dszB-F	ATGACAAGCCGCGTCGACCCCGCAAAC	*dszB*	1098
dszB-R	CTATCGGTGGCGATTGAGGCTGTTG		
dszC-F	ATGACACTGTCACCTGAAAAGCAGC	*dszC*	1254
dszC-R	TCAGGAGGTGAAGCCGGGAATCG		
dszD-F	TTGTCTGACAAGCCGAATGCCGTTTC	*dszD*	579
dszD-R	CTATTGACCTAACGGAGTCGGGC		
27F	AGAGTTTGATCMTGGCTCAG	*16S rRNA gene*	1600
1492R	TACGGYTACCTTGTTACGACTT		

Table S2. BDS volumetric activity and total productivity values during growth studies at different carbon sources (Figure 3 in the manuscript).

Strain	Carbon source	Growth time (h)	Volumetric BDS Activity (U/L)	Total BDS Productivity (U/L/h)	% Increase compared to IGTS8
Rhodococcus qingshengii IGTS8	Glucose	19	-	-	-
		35	-	-	-
		48	-	-	-
		71	-	-	-
	Glycerol	19	2595 ± 243	136.6 ± 12.8	-
		35	3544 ± 174	101.3 ± 5.0	-
		48	8659 ± 708	180.4 ± 14.8	-
		71	11541 ± 1568	162.6 ± 22.1	-
	Ethanol	19	2896 ± 480	152.4 ± 25.3	-
		35	9516 ± 398	271.9 ± 11.4	-
		48	16598 ± 1602	345.8 ± 33.4	-
		71	9041 ± 2620	127.3 ± 25.7	-
Rhodococcus qingshengii ATHUBA4003	Glucose	19	-	-	-
		35	-	-	-
		48	-	-	-
		71	-	-	-
	Glycerol	19	2455 ± 240	129.2 ± 12.6	-5.39
		35	5202 ± 186	148.6 ± 5.3	46.8
		48	11193 ± 249	233.2 ± 5.2	29.3
		71	13012 ± 699	183.3 ± 9.9	12.8
	Ethanol	19	7212 ± 550	379.6 ± 28.9	149.0
		35	14471 ± 1159	413.46 ± 33.1	52.1
		48	18774 ± 918	391.1 ± 19.1	13.1
		71	10362 ± 676	145.9 ± 9.5	14.6
Rhodococcus qingshengii ATHUBA4006	Glucose	19	-	-	-
		35	-	-	-
		48	-	-	-
		71	-	-	-
	Glycerol	19	3900 ± 282	205.6 ± 14.9	50.3
		35	4134 ± 239	118.1 ± 6.8	16.7
		48	7268 ± 237	151.4 ± 4.9	-16.2
		71	10126 ±708	142.6 ± 10.0	-12.3
	Ethanol	19	7950 ± 901	418.4 ± 47.4	174.5
		35	16338 ± 649	466.8 ± 18.5	71.7
		48	21330 ± 1968	444.4 ± 41.0	28.5
		71	16917 ± 120	238.3 ± 16.9	87.1

Table S3. BDS volumetric activity and total productivity values during growth studies at different sulfur sources (Figure 4 in the manuscript).

Strain	Sulfur source	Growth time (h)	Volumetric BDS Activity (U/L)	Total BDS Productivity (U/L/h)	% Increase compared to IGTS8
Rhodococcus qingshengii IGTS8	DMSO	22.5	2027 ± 227	90.1 ± 4.0	-
		39.5	10005 ± 841	253.3 ± 6.4	-
		45	11521 ± 269	256.0 ± 5.7	-
		69	12520 ± 238	181.5 ± 2.6	-
	$DMSO_2$	22.5	454 ± 98	20.2 ± 4.4	-
		39.5	7184 ± 672	181.9 ± 17.0	-
		45	10704 ± 369	237.9 ± 8.2	-
		69	1834 ± 263	26.6 ± 3.8	-
	Taurine	22.5	395 ± 74	17.6 ± 3.3	-
		39.5	12207 ± 609	309.0 ± 15.4	-
		45	14790 ± 849	328.7 ± 18.9	-
		69	4931 ± 543	71.5 ± 7.9	-
Rhodococcus qingshengii ATHUBA4003	DMSO	22.5	6711 ± 551	298.3 ± 24.5	231
		39.5	18923 ± 1582	479.1 ± 40.1	89
		45	20175 ± 2382	448.3 ± 52.9	75
		69	10713 ± 846	155.3 ± 12.3	-14
	$DMSO_2$	22.5	4093 ± 21	181.9 ± 9.3	800
		39.5	10288 ± 1438	260.5 ± 36.4	43
		45	15917 ± 1344	353.7 ± 29.9	49
		69	1424 ± 72	20.6 ± 1.1	-22
	Taurine	22.5	3091 ± 445	137.4 ± 19.8	683
		39.5	14708 ± 530	372.4 ± 13.4	20
		45	21517 ± 1880	478.2 ± 41.8	45
		69	3552 ± 281	51.8 ± 4.1	-28
Rhodococcus qingshengii ATHUBA4006	DMSO	22.5	9418 ± 643	418.6 ± 28.6	365
		39.5	20673 ± 2379	523.4 ± 60.2	107
		45	25161 ± 1835	559.1 ± 40.8	118
		69	14474 ± 878	209.8 ± 12.7	16
	$DMSO_2$	22.5	5320 ± 306	236.5 ± 13.6	1069
		39.5	15051 ± 1594	381 ± 40.4	11
		45	19999 ± 1340	444.4 ± 29.8	87
		69	6202 ± 620	9.2 ± 0.3	-65
	Taurine	22.5	4432 ± 101	197.0 ± 4.5	1022
		39.5	18751 ± 701	474.7 ± 17.8	54
		45	25405 ± 344	564.6 ± 7.7	72
		69	4095 ± 141	59.4 ± 2.1	-17

Table S4. BDS volumetric activity and total productivity values during growth studies at different nitrogen sources (Figure 5 in the manuscript).

Strain	Nitrogen Source	Growth time (h)	Volumetric BDS Activity (U/L)	Total BDS Productivity (U/L/h)	% Increase compared to IGTS8
Rhodococcus qingshengii IGTS8	NH_4Cl	23	3650 ± 429	154.8 ± 18.7	-
		40	13568 ± 741	339.2 ± 18.5	-
		50	15196 ± 1573	303.9 ± 31.5	-
		67	10169 ± 885	151.8 ± 13.2	-
	NH_4NO_3	23	2203 ± 625	95.8 ± 27.2	-
		40	8022 ± 1273	200.6 ± 31.8	-
		50	7638 ± 844	152.8 ± 16.9	-
		67	3975 ± 360	59.3 ± 5.4	-
	$NaNO_3$	23	-	-	-
		40	-	-	-
		50	-	-	-
		67	-	-	-
	Urea	23	4182 ± 116	181.8 ± 5.1	-
		40	10076 ± 333	251.9 ± 8.3	-
		50	9246 ± 634	188.5 ± 12.6	-
		67	10414 ± 647	155.4 ± 9.7	-
Rhodococcus qingshengii ATHUBA4003	NH_4Cl	23	9892 ± 370	430.1 ± 16.1	178
		40	21069 ± 885	526.7 ± 22.1	55
		50	17858 ± 53	357.2 ± 1.1	18
		67	13932 ± 198	207.9 ± 2.9	37
	NH_4NO_3	23	4839 ± 328	210.4 ± 14.3	119
		40	15602 ± 2630	390.1 ± 65.8	94
		50	14139 ± 185	282.8 ± 3.7	85
		67	9276 ± 338	138.5 ± 0.5	133
	$NaNO_3$	23	5159 ± 117	224.3 ± 5.1	-
		40	12138 ± 185	303.5 ± 4.6	-
		50	13139 ± 110	262.8 ± 2.2	-
		67	9191 ± 969	137.2 ± 14.5	-
	Urea	23	5586 ± 1095	242.9 ± 47.6	34
		40	13575 ± 257	339.4 ± 6.5	35
		50	18179 ± 219	363.6 ± 4.4	93
		67	10976 ± 1308	163.8 ± 19.5	5

Continued on next page

Strain	Nitrogen Source	Growth time (h)	Volumetric BDS Activity (U/L)	Total BDS Productivity (U/L/h)	% Increase compared to IGTS8
Rhodococcus qingshengii ATHUBA4006	NH_4Cl	23	11096 ± 656	482.5 ± 28.5	212
		40	20672 ± 872	516.8 ± 21.8	52
		50	17570 ± 905	351.4 ± 18.1	16
		67	8568 ± 416	127.9 ± 6.2	-15
	NH_4NO_3	23	6288 ± 844	273.4 ± 36.7	185
		40	17464 ± 552	436.6 ± 13.8	118
		50	17569 ± 1077	351.4 ± 21.6	130
		67	11467 ± 740	171.2 ± 11.1	188
	$NaNO_3$	23	5287 ± 445	229.9 ± 19.4	-
		40	9499 ± 219	237.5 ± 5.5	-
		50	11208 ± 314	224.2 ± 6.3	-
		67	16957 ± 1594	253.1 ± 23.8	-
	Urea	23	9882 ± 995	429.7 ± 43.3	136
		40	21541 ± 708	538.5 ± 17.7	114
		50	20082 ± 1472	401.7 ± 29.4	113
		67	19414 ± 660	289.8 ± 9.9	86

AIMS Press

© 2022 the Author(s), licensee AIMS Press. This is an open access article distributed under the terms of the Creative Commons Attribution License (http://creativecommons.org/licenses/by/4.0)
